# Notes on a Case of Poisoning from Mrs. Winslow’s Soothing Syrup

**Published:** 1884-12-20

**Authors:** A. B. Hirsh


### NOTES ON A CASE OF POISONING FROM MRS. WINSLOW'S SOOTHING SYRUP.

Read before the Philadelphia County Medical Society, September 17, 1884, by A. B. HIRSH, M. D.

With the object of adding my quota to the list of serious accidents resulting from the indiscriminate sale of secret medical preparations, I gathered the notes of the following case:

Mrs. A. H. L. took her 20-month-old boy to visit some friends, and, while there, they (all unknown to her) fed him some unpealed apple and other indigestible material. Being colicky all that night and next morning, she was pursuaded by a "friend" to purchase a two-ounce vial of the nostrum sold as "Mrs. Winslow's Soothing Syrup," and of this gave him half-teaspoonful doses, as the directions called for, although she insists half of each quantity was spilled through his struggling.

He took, therefore, the first dose at 4 o'clock on Sunday afternoon (August 24), and, there being no effect, another at 8; then dozing but not sleeping from this time till 3 next morning, the pain starting him whining, he was dosed at 5; still crying on, three-quarters of an hour later the final similar amount was administered.

The mother soon became alarmed at the marked stupor which had now set in. He would touch none of the breakfast placed before him, Mrs. L. said : although sitting upright in his high chair, his head hung listlessly, and he recognized nobody.

I saw him at 7.45 a. m., and found marked symptoms present of poisoning by some narcotic drug. The pupil was contracted down to the typical pinhead ; stupor was unmistakable ; respiration was very slow, while at irregular intervals he would take two or three rapidly succeeding deep sighs ; the pulse was rapid and small ; the extremities were cold throughout the case. Taking all these symptoms into consideration, and the fact that the breath bore the peculiar odor of an opiate, I felt warranted in treating the case for one of poisoning by some preparation or derivative of that drug.

The stomach and bowels were emptied at once ; frequent cold sponging was ordered, with wet cloths placed on the nape of the neck whenever great trouble existed in keeping him awake. Tr. belladonna was given hourly in aqueous solution. The parents were directed to keep him awake, by all means.

By noon he would begin to lift the eyelids a little. but relapsed into a sort of dose by 2 p. m. Despite all their efforts, he fell once more into stupor by 6. Calling about this time, I insisted on the mechanical exercises being continued, feeling encouraged by the somewhat improved breathing, and that I succeed a little while later in arousing him. As the pupil had now slowly begun to dilate, the medicine was ordered to be given every half hour, or twice as often as before. By 11 he began to lighten up, and, on calling half an hour later, I found him languidly trying to push his ball around the table upon which he sat ; the pupils were widely dilated. and respiration free. He was allowed to sleep, with slight interruptions, from midnight until 6 a. m., after which the child showed his great thirst by frequent demands for ice-water. Incoordination of the voluntary muscles now became noticeable, and continued until next morning. A typical belladona rash was now likewise beautifully shown, also to disappear in time. He slept for two hours, about noon, being exceedingly irritable afterwards; but, excepting the use of a tonic, required no other treatment.

As stated in the beginning of the notes, this case is merely placed on record to help expose an existing evil, believing that continuous agitation will finally induce the intelligent public to demand the regulation of the sale of patent medicines, a fact concerning which there never was any doubt in the profession.

A fatal result would inevitably have occurred had no treatment been instituted, and I feel convinced that many such cases happen in our midst, which should be reported. Incidentally conversing with both Drs. Schoales and Blackwood, I heard of such occurring in their practices, and should be glad to hear more fully of them from the gentlemen.

The case is the more pertinent at this time, when any fakir or shopkeeper may legally retail unlabeled poisons in the guise of patent medicines, while one of our inconsistent laws is now being so interpreted as to inform the patient that, in very many cases, his doctor has prescribed him medicine containing poison.—*New Orleans Med. and Surg. Journal.*

